# Elevated Alanine Transaminase-to-Platelet Index (APRI) Is Associated with Obesity and Distinct Forms of Dyslipidemia: A Retrospective Cross-Sectional Study

**DOI:** 10.3390/jcm13185650

**Published:** 2024-09-23

**Authors:** Yazeed Alshuweishi, Dalal Alfayez, Abdulmalik A. Almufarrih, Arwa Abudawood, Hanan Alyami, Faisal A. Alshuweishi, Yazeed A. Al-Sheikh, Mohammad A. Alfhili

**Affiliations:** 1 Chair of Medical and Molecular Genetics Research, Department of Clinical Laboratory Sciences, College of Applied Medical Sciences, King Saud University, Riyadh 12372, Saudi Arabiamalfeehily@ksu.edu.sa (M.A.A.); 2 Department of Family and Community Medicine, Prince Sultan Military Medical City, Riyadh 11159, Saudi Arabia; 3 Department of Medical and Surgical Nursing, College of Nursing, Princess Norah bint Abdurrahman University, Riyadh 11564, Saudi Arabia; 4 Department of Pathology and Laboratory Medicine, King Khalid University Hospital, King Saud University Medical City, Riyadh 12372, Saudi Arabia

**Keywords:** obesity, APRI score, dyslipidemia, inflammation, ALT, platelets

## Abstract

**Background:** Obesity is a pathological condition and a major risk factor for dyslipidemia, type 2 diabetes, and non-alcoholic fatty liver disease. Recent research highlighted the association of non-invasive serum markers with these conditions but the clinical utility of ALT APRI in obesity and its relationship with dyslipidemia remain unexplored. **Methods:** We examined the association of ALT APRI in 165 non-diabetic adults stratified by BMI and serum lipid parameters. **Results:** Obese subjects had significantly higher APRI than lean subjects, with an area under the curve (AUC) of 0.65 (*p* = 0.019). Medians of APRI were significantly increased in subjects with high TG, TG/HDL, TC/HDL, and LDL/HDL and low HDL. Notably, all lipid parameters and ratios were significantly elevated in the highest APRI tertile, compared with patients in the lowest tertile. APRI was weakly yet significantly correlated with BMI (R^2^ = 0.032, *p* = 0.022), HDL (R^2^ = 0.071), TG/HDL (R^2^ = 0.031), TC/HDL (R^2^ = 0.063), LDL/HDL (R^2^ = 0.072), and TyG index (R^2^ = 0.081). While APRI only showed a discriminating capacity for HDL (AUC: 0.69, *p* = 0.003), TG/HDL (AUC: 0.63, *p* = 0.020), LDL/HDL (AUC: 0.68, *p* < 0.001), and TyG index (AUC: 0.65, *p* = 0.037), the highest diagnostic performance of APRI was observed with TC/HDL (AUC: 0.74, *p* < 0.001). Additionally, APRI was a risk factor for high TG (OR: 1.6, *p* = 0.028), low HDL (OR: 2.7, *p* = 0.0002), high TG/HDL (OR: 1.94, *p* = 0.0011), high TC/HDL (OR: 2.3, *p* < 0.0001), high LDL/HDL (OR: 2.2, *p* = 0.0001), and high TyG index (OR: 2.1, *p* = 0.008). **Conclusions:** Our findings argue for the role of APRI as a potential marker for obesity and dyslipidemia, which requires further confirmation in longitudinal studies.

## 1. Introduction

Obesity has escalated to epidemic proportions on a global scale, with a marked increase in prevalence observed over the past few decades. According to the World Health Organization (WHO), the prevalence of obesity between 1975 and 2016 increased globally by over three times [1]. In 2016 alone, the WHO reported that over 1.9 billion adults were categorized as overweight, among which 650 million were classified as obese [2]. The increased prevalence of obesity is not confined to high-income countries; it is also increasingly observed in low- and middle-income nations [3]. The multifactorial etiology of obesity comprises genetic, environmental, and behavioral components, necessitating a multidimensional approach to its prevention and management.

Obesity is defined as increased adipose tissue expansion, either in the form of hypertrophy (enlargement of existing fat cells) or hyperplasia (increase in the number of fat cells). This expansion can disrupt metabolic homeostasis, leading to an elevated risk of metabolic disorders such as type 2 diabetes (T2D) and non-alcoholic fatty liver disease (NAFLD) [4,5,6]. Obesity is associated with abnormal lipid metabolism including elevated triglyceride (TG), low-density lipoproteins (LDL), and lowered high-density lipoproteins (HDL) and apolipoprotein A-I (ApoA-I) [7,8,9]. The extent of lipid abnormalities is associated with body mass index (BMI), with 60–70% of obese individuals and 50–60% of those who are overweight experiencing dyslipidemia [7]. Dyslipidemia observed in obesity is mainly driven by different abnormalities including insulin resistance, adiposity-induced proinflammatory state, and increased hepatic fatty acid uptake [10,11,12]. In obese patients, reduced insulin activity due to insulin resistance results in elevated TG lipolysis in adipose tissue leading to an increased influx of fatty acid to the liver [13]. Over time, this increase in fatty acid delivery to the liver leads to enhanced hepatic fatty acid synthesis and subsequent very low-density lipoprotein (VLDL) secretion, which is an important contributor to the elevation of serum TG levels [14,15]. Given the asymptomatic nature of dyslipidemia, routine screening becomes essential for its early detection to provide an opportunity for significant reductions in morbidity and mortality. Utilizing early biomarkers and other risk assessment tools is crucial for healthcare providers, as it enables them to implement tailored interventions that can significantly mitigate these risks.

The liver is a key organ in metabolism, crucial for regulating carbohydrates, lipids, and proteins [16,17]. In lipid metabolism, the liver is responsible for synthesizing, oxidizing, and storing fatty acids, as well as producing and secreting lipoproteins that transport lipids throughout the body [16,17]. In hepatocytes, only small amounts of fatty acids are stored as triglycerides while those remaining are eliminated by oxidation within the cell or by secretion into the circulation in TG-rich VLDL [16,17]. In the context of obesity, hepatic fatty acid metabolism is disrupted, leading to the accumulation of triglycerides within hepatic tissue and resulting in a clinical condition known as non-alcoholic fatty liver disease (NAFLD) [18,19]. Recently, a redefinition of this disease to metabolic-associated fatty liver disease (MAFLD) has been proposed to more accurately reflect its underlying pathophysiology [20]. This new terminology emphasizes the central role of metabolic dysfunctions such as insulin resistance, dyslipidemia, and systemic inflammation in the development and progression of fatty liver disease. Given the role of the liver in lipid metabolism, it is not surprising that several markers of liver injury, including aspartate transferase (AST), alanine aminotransferase (ALT), alkaline phosphatase (ALP), and γ-glutamyl-transferase (GGT) have been associated with obesity and the risk of developing metabolic syndrome [21,22,23]. Analysis of a population from the National Health and Nutrition Examination Survey (NHANES) database revealed that multiple liver enzymes including ALT, AST, ALP, and GGT were significantly increased as degrees of general obesity increased [24]. Furthermore, ALT was strongly associated with the prevalence of metabolic syndrome in 236 children and adolescents among Mexican children [25]. Notably, ALT, among other liver enzymes, has been consistently linked to obesity and metabolic syndrome, as reported in numerous studies [26,27,28,29].

Recently, non-invasive liver fibrosis scores such as the AST-to-platelet ratio index (APRI) and the fibrosis-4 (FIB-4) have emerged as serum biomarkers in multiple hepatic diseases [30,31,32,33,34]. Notably, the APRI score is widely used to assess liver fibrosis and cirrhosis, particularly in patients with chronic liver diseases and it is calculated using serum aspartate aminotransferase (AST) levels and platelet counts, providing a cost-effective alternative to liver biopsy [35,36,37]. Similarly, indices employing alternative liver enzymes–alanine aminotransferase (ALT), gamma-glutamyl transferase (GGT), and alkaline phosphatase (ALP), respectively, in conjunction with platelet counts, were utilized as additional non-invasive assessments of liver function and fibrosis. These include the ALT-to-platelet ratio index (ALT APRI), the gamma-glutamyl transferase-to-platelet ratio (GPR), and the alkaline phosphatase-to-platelet ratio (APPRI) [31,38,39]. We have lately shown that the ALT APRI shows better diagnostic ability in identifying hyperglycemia than the AST APRI score [39]. However, the clinical utility of the ALT APRI score in obesity and its association with metabolic parameters has been unexplored. Given the significant prevalence of obesity and its associated metabolic complications, including dyslipidemia, discovering potential biomarkers is crucial in the fight against obesity and its complications to allow for early intervention and prevent or delay the onset of such complications. Since the ALT APRI score is a feasible and easily calculated marker, this study aims to investigate the patterns of ALT APRI scores in obese patients and its association estimates, risk assessment, and diagnostic accuracy with individual lipid parameters.

## 2. Methods

### 2.1. Study Design and Data Collection

For this retrospective study, data were collected between 2022 and 2023 from the Family Medicine Booked Clinic and Family Medicine Lifestyle Clinic at the Prince Sultan Military Medical City (PSMMC), Riyadh, Saudi Arabia. The Institutional Review Board (IRB) of PSMMC provided ethical clearance (IRB number: E-2165; approved on 14 September 2023). Patients were excluded if they were younger than 18 years of age, pregnant, or were diagnosed with diabetes or liver disease. The studied subjects were classified according to BMI into three classes, with BMI from 18 to 24.9 kg/m^2^ as normo-weight, from 25 to 29.9 kg/m^2^ as overweight, and 30 kg/m^2^ or greater as obese [40]. The *ALT APRI* score was calculated as follows:

$$ALT\;APRI=\left(\frac{ALT}{ULN\;of\;ALT}\right)\times \frac{100}{platelet\;count}$$

The upper limit of normal (*ULN*) for *ALT* used in this study was 40 U/L [41]. Dyslipidemia was identified in individuals who had lipid profiles with the following abnormalities: TC ≥ 200 mg/dL, LDL ≥ 130 mg/dL, HDL < 40 mg/dL, and TG of ≥150 mg/dL [41]. Accordingly, TC/HDL ratio ≥ 5, LDL/HDL ratio ≥ 3, and TG/HDL ratio ≥ 3 were considered high. The *TyG index* (triglyceride-glucose index) is a lipid-related ratio and was included in this study due to its emerging relevance in assessing insulin resistance and metabolic health. A *TyG index* > 4.72 was considered high [42] and was calculated using the following formula:

$$TyG\;index=\frac{TG\times FBG}{2}$$

### 2.2. Data Collection

Data were retrieved from electronic medical records with all information was anonymized to maintain patient confidentiality. The following variables were gathered from each subject:

1. Demographic and clinical data: Age, sex, and BMI were recorded. Patients were advised to remove shoes and heavy clothing during the weight assessment. Weight and height were measured using a weighing scale and a portable stadiometer (Marsden H226, Marsden Weighing Group, South Yorkshire, UK). To calculate BMI, body weight (in kilograms) was divided by body height (in square meters). Medical history and current medications were reviewed to ensure adherence to the inclusion criteria.
2. Laboratory Data: Blood samples were collected routinely following established protocols and sent to a central laboratory. Regular quality assurance and control checks were performed on all laboratory equipment. Fasting blood glucose (FBG), triglycerides (TG), total cholesterol (TC), high-density lipoprotein (HDL), and low-density lipoprotein (LDL) levels were analyzed using a Cobas-8000 autoanalyzer (Roche Diagnostics, Rotkreuz, Switzerland). Hemoglobin A1c (HbA1c) levels were determined with a Cobas-513 autoanalyzer (Roche Diagnostics, Rotkreuz, Switzerland).

### 2.3. Statistical Analysis

As revealed by the D’Agostino and Pearson test and the Kolmogorov–Smirnov test (*p* < 0.0001), the collected data were not normally distributed and thus nonparametric tests were used. The Kruskal–Wallis test was used to compare three study groups while the Mann–Whitney U test was used to compare two study groups. The analyzed data were displayed as medians ± interquartile range (IQR). A correlation analysis between lipid parameters and APRI score was assessed by simple linear regression analysis, and risk assessment was determined by calculations of the prevalence risk (PR) and odds ratio (OR). The diagnostic accuracy of the APRI score to discriminate high BMI and lipid abnormalities was assessed by ROC curve analysis and area under the curve (AUC). The statistical analysis was performed using GraphPad Prism v9.2.0 (GraphPad Software, Inc., San Diego, CA, USA), and statistical significance was set at *p* < 0.05.

## 3. Results

### 3.1. Baseline Characteristics of the Studied Population

A total of 165 patients (46 males and 119 females) were included in this retrospective study. The median BMI was 33 kg/m^2^ (±27–37) and the median age was 37 years (±28–45). The clinical and biochemical data are shown in Table 1 and Table 2, respectively. Compared to normo-weight and overweight subjects, obese patients exhibited increased RBC, WBC, lymphocyte, and monocyte counts. No changes were seen in the levels of hemoglobin, hematocrit, mean corpuscular volume (MCV), and mean corpuscular hemoglobin (MCH) between all groups. Fasting blood glucose (FBG) and hemoglobin A1C (HbA1C) were significantly higher while albumin concentration was lower in the obese groups, compared to normo-weight and overweight groups.

### 3.2. APRI Score Is Significantly Elevated in Obese Subjects and Showed a Better Diagnostic Accuracy for Obesity

**Figure 1:**
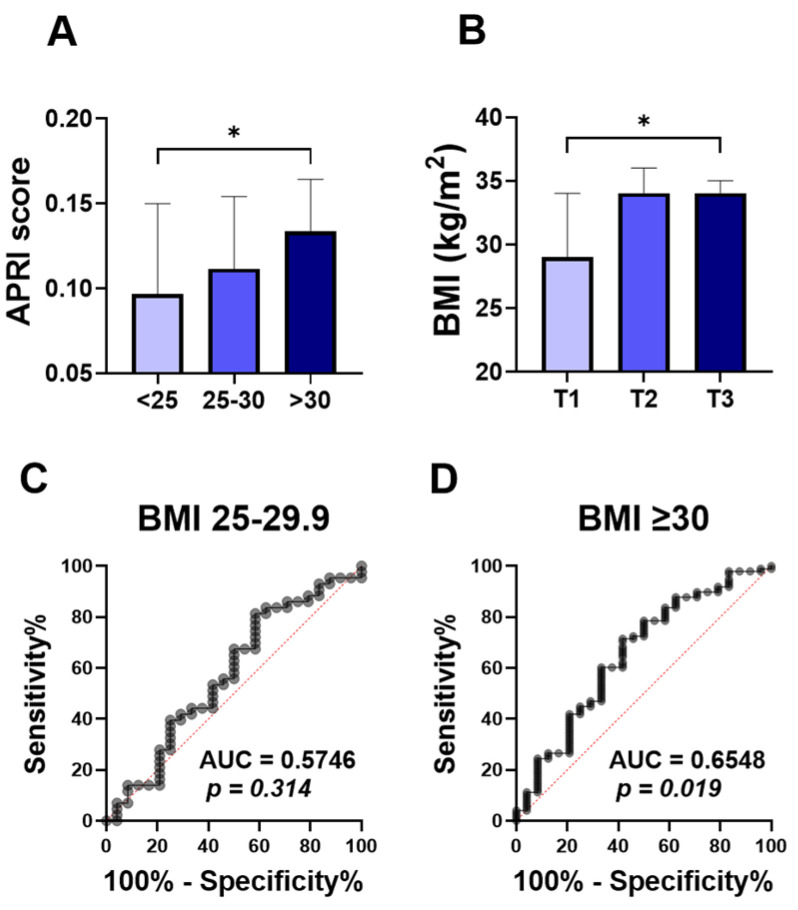
Comparison of APRI score among BMI classification and ROC curve of APRI score for obesity. Medians ± IQR of APRI values among normal weight (<25), overweight (25–29), and obese subjects (≥30) (**A**). Medians ± IQR of BMI values among the first tertile (T1), the second tertile (T2), and the third tertile (T3) (**B**). ROC curves of the elevated APRI and BMI between 25–30 (**C**), and BMI above 30 (**D**). * indicates (*p* < 0.05) significance.

To assess the relationship between the ALT APRI score and obesity, we compared APRI score values in normo-weight, overweight, and obese groups. The APRI score was significantly higher in the obese patients (Figure 1A; 0.133 ±0.100–0.239), compared to the normo-weight group (Figure 1A; 0.097 ± 0.069–0.157). No significant differences were found in the level of APRI score when overweight subjects (Figure 1A; 0.111 ± 0.086–0.175) were compared to the other groups. To further examine this, the studied population was stratified by APRI score tertiles. With 55 patients in each group, the first tertile (T1) had APRI scores below 0.1004, the second tertile (T2) ranged from 0.1004 to 0.1640, and the third tertile (T3) had scores above 0.1640. Notably, the higher APRI score tertile (T3) showed a significant increase in the BMI degree (Figure 1B; 34 ± 28–38), compared to the T1 tertile group (Figure 1B; 29± 25–36).

The ROC curve for diagnostic accuracy of the APRI score in differentiating obesity is analyzed in Figure 1C,D. The diagnostic accuracy of the APRI score in discriminating overweight was not significant, with the area under the curve (Figure 1C; AUC = 0.57, *p* = 0.314) reflecting the poor diagnostic accuracy of APRI scores to distinguish between normo-weight and overweight groups. However, the APRI score showed a better diagnostic accuracy for obesity (Figure 1D; AUC = 0.65, *p* = 0.019).

### 3.3. APRI Score Is Significantly Increased in Elevated TG and All Lipid Ratios

**Figure 2:**
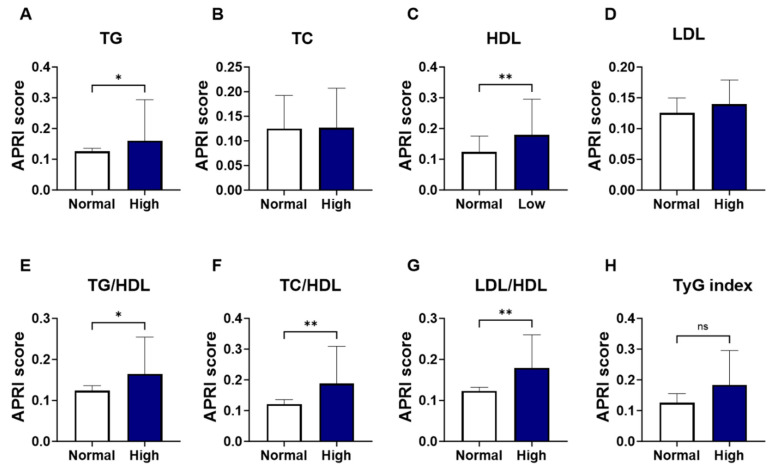
Changes in APRI score in light of different lipid parameters. Medians ± IQR of APRI values among individual lipid marker TG (**A**), TC (**B**), HDL (**C**), LDL (**D**), TG/HDL (**E**), TC/HDL (**F**), LDL/HDL (**G**), and TyG index (**H**). ns indicates not significant while * (*p* < 0.05), and ** (*p* < 0.01).

As shown in Figure 2, groups were formed based on the normal and abnormal levels of the individual marker of lipid profile, and the APRI score values were analyzed. Levels of APRI scores were significantly increased in patients with high TG (Figure 2A; 0.160 ± 0.08–0.19 vs. 0.125 ± 0.09–0.18, *p* = 0.037) and low HDL patients (Figure 2C; 0.179 ± 0.12–0.31 vs. 0.123 ± 0.08–0.18, *p* = 0.005), compared to the normal-counterpart groups. No significant alterations were seen in the level of APRI score patients with high TC (Figure 2B; 0.127 ± 0.10–0.21 vs. 0.125 ± 0.08–0.19, *p* = 0.258) or LDL (Figure 2D; 0.140 ± 0.10–0.26 vs. 0.125 ± 0.08–0.18, *p* = 0.154). The increase in APRI score was clearly apparent in the high lipid ratios including high-TG/HDL (Figure 2E; 0.164 ± 0.11–0.28 vs. 0.124 ± 0.08–0.18, *p* = 0.016) high-TC/HDL (Figure 2F; 0.188 ± 0.13–0.34 vs. 0.121 ± 0.08–0.17, *p* = 0.001), and high-LDL/HDL (Figure 2G; 0.179 ± 0.12–0.30 vs. 0.123 ± 0.08–0.17, *p* = 0.002). However, when patients were grouped based on TyG index levels, a trend toward an increase in APRI score level was observed in high-TyG patients, yet this did not achieve statistical significance (Figure 2H; 0.184 ± 0.12–0.30 vs. 0.126 ± 0.08–0.19, *p* = 0.09).

### 3.4. The Highest Tertile of APRI Score Exhibits All Forms of Dyslipidemia

**Figure 3:**
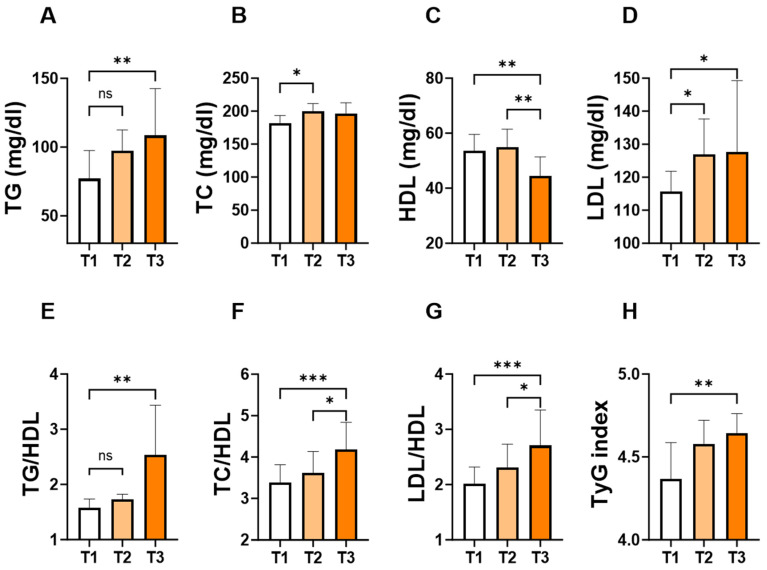
Levels of lipid parameters and ratios based on the tertiles of APRI score. Medians ± IQR of TG (**A**), TC (**B**), HDL (**C**), LDL (**D**), TG/HDL (**E**), TC/HDL (**F**), LDL/HDL (**G**), and TyG index (**H**) according to APRI score tertiles: T1 (<0.1004), T2 (0.1004–0.1640) and T3 (>0.1640). ns indicates not significant while * (*p* < 0.05), ** (*p* < 0.01), and *** (*p* < 0.001).

To assess the changes in individual lipid markers and ratios in light of the APRI score, patients were stratified into tertiles based on the levels of the APRI score. As shown in Figure 3, the concentration of TG (Figure 3A; 109 ± 87–162 vs. 77 ± 63–119, *p* = 0.007) and LDL (Figure 3D; 128 ± 105–154 vs. 116 ± 89–129, *p* = 0.03) concentrations were significantly higher in T3 groups compared to their counterpart T1 groups. Conversely, T3 showed the lowest level of HDL (Figure 3C; 44 ± 37–54 vs. 54 ± 45–61, *p* = 0.005) compared to T1 or T2. Furthermore, TG/HDL (Figure 3E; 2.5 ± 1.5–4.3 vs. 1.6 ± 1.1–2.1, *p* = 0.002), TC/HDL (Figure 3F; 4.2 ± 3.5–5.8 vs. 3.4 ± 2.9–4.0, *p* = 0.0003), LDL/HDL (Figure 3G; 2.7 ± 2.3–3.7 vs. 2.0 ± 1.7–2.6, *p* = 0.0001), and TyG index (Figure 3H; 4.6 ± 4.5–4.8 vs. 4.4 ± 4.3–4.6, *p* = 0.01) showed a significant increase in the T3 group compared to their counterpart T1 groups.

### 3.5. APRI Score Is Differentially Correlated with Lipid Markers

**Figure 4:**
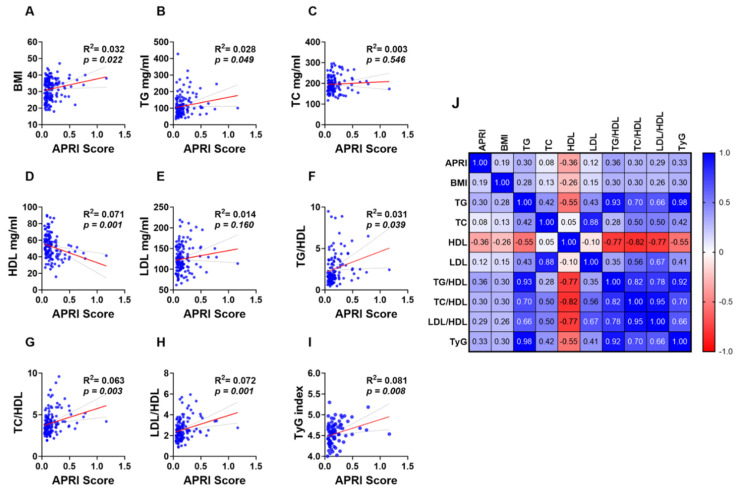
Association of APRI with lipid parameters and ratios in the studied population. Simple linear regression of the association of APRI with BMI (**A**), TG (**B**), TC (**C**), HDL (**D**), LDL (**E**), TG/LDL (**F**), TC/HDL (**G**), LDL/HDL (**H**), and TyG index (**I**). A correlation matrix with correlation coefficients is also shown (**J**).

Simple linear regression analysis revealed a weak yet significant positive and negative correlation between the APRI score and lipid makers and ratios. BMI (Figure 4A; R^2^ = 0.032 and *p* = 0.022) and TG (Figure 4B; R^2^ = 0.028 and *p* = 0.049) showed a positive correlation with the APRI score while a negative correlation was observed between HDL and the APRI score (Figure 4D; R^2^ = 0.071 and *p* = 0.001). However, TC (Figure 4C; R^2^ = 0.003 and *p* = 0.546) and LDL (Figure 4E; R^2^ = 0.014 and *p* = 0.160) showed no significant correlation with the APRI score. Additionally, all lipid ratios including TG/HDL (Figure 4F; R^2^ = 0.031 and *p* = 0.039), TC/HDL (Figure 4G; R^2^ = 0.063 and *p* = 0.003), LDL/HDL (Figure 4H; R^2^ = 0.072 and *p* = 0.001) and TyG index (Figure 4I; R^2^ = 0.081 and *p* = 0.008) were positively correlated with APRI score values as shown in Figure 4F–I.

**Figure 5:**
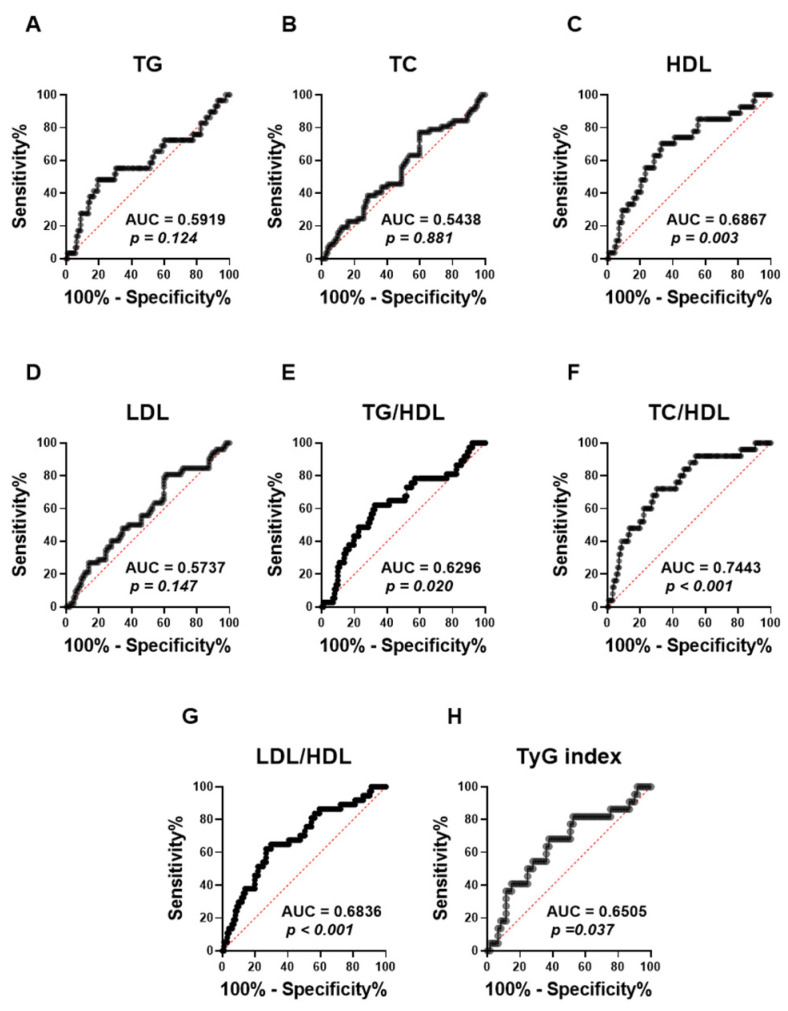
Evaluating the ability of APRI to discriminate different forms of dyslipidemia in the studied population. ROC curves of the elevated APRI and TG (**A**), TC (**B**), HDL (**C**), LDL (**D**), TG/LDL (**E**), TC/HDL (**F**), LDL/HDL (**G**), and TyG index (**H**) are illustrated.

ROC (receiver operating characteristic) curves were performed to assess the capacity of the APRI score to distinguish lipid abnormalities among the studied population (Figure 5). The analysis revealed that the APRI score was not able to distinguish TG (Figure 5A; AUC = 0.59, *p* = 0.124), TC (Figure 5B; AUC = 0.54, *p* = 0.881), and LDL (Figure 5D; AUC = 0.57, *p* = 0.147), as shown by their low AUC values and the lack of statistical significance. By contrast, APRI scores demonstrated a significant discriminatory performance for HDL values (Figure 5C; AUC = 0.69, *p* = 0.003). Moreover, the APRI score showed a better performance in discriminating All high lipid ratios, including TG/HDL (Figure 5E; AUC = 0.63, *p* = 0.020), LDL/HDL (Figure 5G; AUC = 0.68, *p* > 0.001) and TyG index (Figure 5H; AUC = 0.65, *p* = 0.037). Notably, the APRI score showed the highest AUC value with high TC/HDL, as shown in Figure 5F (AUC = 0.74, *p* > 0.001).

### 3.6. High APRI Score Is a Marker of Lipid Abnormalities

To examine the prevalence of all forms of lipid abnormalities relative to the APRI score, a cutoff value with the highest sensitivity and specificity was chosen. The optimal cut-off value for APRI score value was > 0.1533 based on the best AUC values obtained with TC/HDL as revealed by receiver operating characteristics (ROC) curve analysis. Therefore, APRI score value values > 0.1533 were considered high. Table 3 shows that hypertriglyceridemia was prevalent in 44.83% of patients with a normal APRI score (N-APRI score), and this increased to 55.17% in those with a high APRI score (H-APRI score). Similarly, the prevalence of reduced HDL increased from 33.33% in N-APRI score subjects to 66.67% in H-APRI score subjects. Moreover, the prevalence of abnormal levels of TG/HDL, TC/HDL, LDL/HDL, and TyG index in patients with H-APRI scores were 59.46%, 72%, 63.89%, and 73.68%, respectively.

As shown in Table 4, risk assessment analysis revealed that elevated APRI score values were a risk factor for all abnormal lipid markers except TC and LDL. In particular, the highest PR values were observed with TC/HDL (PR = 4.48, *p* < 0.0001) followed by LDL/HDL (PR = 3.94, *p* = 0.0001) and HDL (PR = 3.77, *p* = 0.0002). Similarly, OR values revealed that the likelihood of having an H-APRI score was significantly higher with low HDL (OR = 3.16, *p* = 0.0016) and high TG/HDL (OR = 3.01, *p* = 0.0026), TC/HDL (OR = 3.54, *p* = 0.0004), LDL/HDL (OR = 3.81, *p* = 0.0004), and TyG index (OR = 2.76, *p* = 0.0058).

## 4. Discussion

Obesity is associated with dyslipidemia and is an important risk factor for several metabolic diseases including type 2 diabetes (T2D), non-alcoholic fatty liver disease (NAFLD), and cardiovascular diseases (CVDs) [43,44]. Early detection of dyslipidemia is imperative, especially for at-risk populations such as obese patients. A new serum-derived biomarker has gained interest in recent years due to its feasibility, low cost, and high availability. AST and ALT APRI scores have been reported to be potential prognostic indicators of non-hepatic conditions such as dysglycemia and metabolic syndrome [39,45].

This study aimed to validate, in a cohort of non-diabetic patients with no previous liver diseases, the correlation between ALT APRI scores and obesity and the role of elevated APRI as a marker of severe obesity-related metabolic disturbances. We demonstrated here, for the first time, that the ALT APRI score is significantly increased in obese patients, compared to subjects with normal weight. Stratified by APRI score, our study showed that subjects with elevated APRI scores had significantly higher BMI levels and exhibited different forms of dyslipidemia. Notably, lower levels of HDL and higher levels of TG, LDL, TC/HDL, TG/HDL, LDL/HDL, and TyG index were observed in elevated APRI-score patients. Except for TC and LDL, all other lipid parameters were significantly correlated with the ARPI score. Particularly, the highest diagnostic accuracy of the APRI score was observed in elevated TC/HDL, as revealed in the ROC curve analysis. These findings reinforce the utility of APRI as a marker of severe obesity-related dyslipidemia, providing evidence to support the use of APRI in clinical practice for identifying individuals at higher risk of dyslipidemia, thereby potentially aiding in the stratification and management of obese patients.

The central finding in this study is the significant association between APRI scores and BMI degrees. This observation points to the potential role of ALT, a component of the APRI score, on the relationship of the APRI score with BMI. ALT is primarily found in the liver, unlike AST, and elevated serum levels of ALT indicate liver cell damage [46]. Numerous studies have documented the correlation between body fat composition and elevated liver enzyme levels [47,48,49]. A study based on a Korean population demonstrated that an increased risk of elevated ALT with higher BMI levels and the likelihood of elevated ALT levels among obese subjects was 5 and 3.9 in men and women, respectively [50]. Among Australian adults, BMI and waist circumference were strongly linked to GGT and ALT levels. Intriguingly, the risk of elevated ALT was about seven times higher in the obese group, while it was only about twice as high in individuals with moderate or heavy alcohol consumption, highlighting the substantial impact of obesity on liver enzyme levels, even more than alcohol consumption, which is already a well-known cause of liver disease [51]. Recently, Ali et al. reported that 58% of patients with general obesity and 55% of patients with abdominal obesity had at least one or more elevated liver enzymes [52]. The changes in serum ALT levels among obese patients may be due to the degree of hepatocyte injury, suggesting obesity-related adverse outcomes in these patients. The most obvious explanation for such a relationship is likely due to ongoing dysglycemia and insulin resistance commonly observed in obesity. Indeed, Xu et al. reported that the association of BMI with dysglycemia was mediated partially by GGT and ALT but not AST, suggesting a role of insulin resistance behind this relationship [53]. Notably, Hanley et al. demonstrated that ALT level was strongly associated with hepatic insulin resistance compared to other liver enzymes, AST and ALP [54]. This might be due to the impact of adipose tissue-derived proinflammatory adipokines, as well as insulin resistance-induced oxidative stress and DNA methylation, causing liver cell damage and an increase in liver enzymes released into the bloodstream [55,56].

It is well-documented that increased liver function test markers are indicative of excessive lipid accumulation in the liver [57,58]. Abnormal levels of TG, LDL, and HDL have been associated with the development of NAFLD while dyslipidemic patients are at higher risk of developing fatty liver disease [57,58]. The prevalence of NAFLD is between 15% and 30% among the general population while it is between approximately 50% and 90% in obese subjects [59]. Additionally, it was shown that hepatic lipid deposition increases approximately by 22%, 21%, and 104% for each 1% increase in total body fat, subcutaneous fat, and abdominal fat, respectively [60]. Furthermore, elevated TG and cholesterol were associated with increased inflammation and ROS generation in liver tissue [61]. Rauchbach et al. demonstrated that cholesterol supplementation to hepatic stellate cells (HSCs) induced oxidative stress, mitochondrial dysfunction, and apoptosis [62]. Our study demonstrated that the highest quartile of APRI score values had the highest levels of TG, TC, and LDL and the lowest levels of HDL, indicating the involvement of liver injury among obese patients. Multiple studies have documented a strong association between elevated ALT activity and hyperlipidemia. For instance, a study performed among a general population in the USA revealed that lipid parameters including LDL, HDL, and TG were found to be good predictors of elevated ALT, and the likelihood of having abnormally higher levels of ALT was significantly increased in subjects with high LDL concentrations [63]. Marchesini et al. reported that ALT activity was associated with hyperlipidemia and insulin resistance among T2DM patients [22]. Similarly, higher BMI, dyslipidemia, and hyperglycemia were found in the highest quartile according to ALT activity [64]. These findings suggest that obesity-associated dyslipidemia may cause hepatocyte injury, mitochondrial dysfunction, and inflammatory cytokine production acting in concert. The combined effect of these mechanisms leads to the persistent elevation of liver function test markers in obese individuals, indicating ongoing liver injury and an increased risk of progression to more severe forms of liver diseases.

Our results demonstrated that the best diagnostic performance by APRI score was observed with increased TC/HDL. Studies have shown that the TC/HDL ratio can be a better predictor of heart disease than individual cholesterol levels, likely due to its comprehensive view of lipid balance and potential risks [65,66,67,68]. Thus, monitoring and managing the TC/HDL ratio is crucial for maintaining cardiovascular health and preventing heart diseases. Given the ability of the APRI score to identify patients with higher TC/HDL levels along with other lipid parameters, it could thus serve as a promising biomarker in combination with TC/HDL for monitoring, assessing, and mitigating the risks associated with cholesterol imbalances. Therefore, it is also necessary for future studies to determine when the measurement of the APRI score is most beneficial in clinical circumstances, and to examine whether it occurs before, at the same time as, or after dyslipidemia.

Our study has several limitations that warrant consideration. The retrospective nature of this study makes it challenging to establish clear cause-and-effect relationships. Additionally, this study included a subset of the obese population with comorbidities such as biliary atresia and hypothyroidism as well as those on weight loss medications like GLP-1 agonists and metformin. These comorbidities and medications could potentially influence the APRI score and lipid profile, thereby introducing variability in our results. This heterogeneity may complicate the direct association between obesity, APRI score, and dyslipidemia. While our study encompasses a clinical context where obese individuals often present with multiple comorbidities and are managed with various medications, they may limit the generalizability of our findings to obese individuals without such comorbidities. Future research should consider stratifying patients based on comorbidity status and medication use to provide a more nuanced understanding of the relationship between obesity, APRI score, and lipid abnormalities.

## 5. Conclusions

In summary, our study demonstrated that the ALT APRI score is elevated in obese patients and strongly associated with dyslipidemia which serves as a crucial biomarker of liver function in the context of obesity. Understanding the relationship between a disturbed lipid profile and the APRI score is essential for early diagnosis and intervention of obesity complications such as NAFLD. Continued research is needed to elucidate the mechanisms driving these changes and to develop targeted therapies for obese patients with liver disease.

## Figures and Tables

**Table 1:** Baseline laboratory characteristics of all subjects included in the study.

	Control (*n* = 24)	Overweight (*n* = 43)	Obese (*n* = 98)	*p* Value
RBCs (×10^12^/μL)	4.6 (±4.3–5.1)	4.7 (±4.4–5.1)	4.9 (±4.5–5.4)	0.0246
Hb (g/dL)	12.95 (±12.0–14.0)	13.10 (±12.2–14.5)	13.25 (±12.1–14.7)	0.2628
Hct L/L	0.39 (±0.38–0.43)	0.41 (±0.38–0.44)	0.41 (±0.38–0.45)	0.2311
MCV (fL)	86 (±81–90)	87 (±83–89)	84 (±80–88)	0.2255
MCH (g/dL)	28.0 (±26.8–29.0)	28.0 (±26.3–29.6)	27.5 (±25.7–29.3)	0.3937
WBCs (×10^9^/μL)	6.7 (±5.0–8.4)	6.0 (±5.0–6.9)	7.3 (±5.5–8.4)	0.0269
Neutrophil (×10^9^/μL)	3.4 (±2.1–5.1)	2.9 (±2.0–3.6)	3.6 (±2.3–4.6)	0.062
Lymphocyte (×10^9^/μL)	2.37 (±1.82–2.58)	2.20 (±2.00–2.70)	2.63 (±2.15–3.12)	0.0082
Monocyte (×10^9^/μL)	0.54 (±0.42–0.63)	0.45 (±0.35–0.53)	0.54 (±0.41–0.65)	0.0087
Eosinophile (×10^9^/μL)	0.14 (±0.10–0.23)	0.15 (±0.09–0.21)	0.18 (±0.11–0.25)	0.3561
Basophile (×10^9^/μL)	0.05 (±0.03–0.06)	0.04 (±0.03–0.06)	0.04 (±0.03–0.06)	0.8548
Platelet (×10^9^/μL)	273.0 (±244–333)	293.0 (±232–338)	299.0 (±254–362)	0.2980
FBG mg/dL	84.7 (±74.3–89.6)	82.9 (±79.3–88.3)	90.1 (±82.9–93.7)	0.0159
HbA1c %	5.5 (±5.2–5.6)	5.4 (±5.2–5.6)	5.7 (±5.5–5.9)	<0.0001
Creatinine µmol/L	65 (±54–77)	62 (±55–73)	64 (±57–79)	0.6393
Albumin g/L	45 (±44–49)	45 (±43–47)	44 (±42–45)	0.0002

Abbreviations: RBC (red blood cells); Hb (hemoglobin); Hct (hematocrit test); MCV (mean corpuscular volume); MCH (mean corpuscular hemoglobin); WBC (white blood cells); FBG (fasting blood glucose); HbA1c (glycated hemoglobin).

**Table 2:** Clinical characteristics and comorbidities of all subjects included in the study.

Variables	Descriptive Statistics
Total number of patients	165
Sex (Female), *n* (%)	119 (72.1%)
Age (y), median (IQR)	37 (±28–45)
BMI, median (IQR)	33 (±27–37)
Weight (kg), median (IQR)	83 (±69–94)
Smoking, *n* (%)	20 (12.1%)
Hypertension, *n* (%)	11 (6.7%)
Biliary atresia, *n* (%)	8 (4.8%)
PCOS, *n* (%)	10 (6.1%)
Hypothyroidism, *n* (%)	23 (13.9%)
IDA, *n* (%)	16 (9.7%)
Infertility, *n* (%)	5 (3.0%)
Iron supplementation, *n* (%)	28 (17%)
GLP-1 agonist, *n* (%)	34 (20.6%)
Metformin, *n* (%)	15 (9.1%)

Abbreviations: BMI, body mass index; PCOS, polycystic ovary syndrome; IDA, iron deficiency anemia; GLP-1, glucagon-like peptide-1.

**Table 3:** Prevalence of different forms of dyslipidemia in light of normal and elevated APRI score.

Parameter	Prevalence (%)
Elevated TG	
Normal APRI < 0.153	44.83
High APRI > 0.153	55.17
Elevated TC	
Normal APRI < 0.153	57.89
High APRI > 0.153	42.11
Reduced HDL	
Normal APRI < 0.153	33.33
High APRI > 0.153	66.67
Elevated LDL	
Normal APRI < 0.153	52.94
High APRI > 0.153	47.06
Elevated TG/HDL	
Normal APRI < 0.153	40.54
High APRI > 0.153	59.46
Elevated TC/HDL	
Normal APRI < 0.153	28.00
High APRI > 0.153	72.00
Elevated LDL/HDL	
Normal APRI < 0.153	36.11
High APRI > 0.153	63.89
Elevated TyG index	
Normal APRI < 0.153	26.32
High APRI > 0.153	73.68

**Table 4:** Risk assessment of obesity and dyslipidemia based on the APRI score.

Parameter	PR	95% CI	Z Statistic	*p*	OR	95% CI	Z Statistic	*p*
TG	1.60	1.05–2.42	2.20	0.0277	2.33	1.02–5.35	2.00	0.0457
TC	1.15	0.76–1.75	0.66	0.509	1.26	0.63–2.51	0.66	0.512
HDL	2.07	1.42–3.03	3.77	0.0002	4.22	1.73–10.31	3.16	0.0016
LDL	1.36	0.91–2.06	1.48	0.1378	1.69	0.83–3.42	1.46	0.1454
TG/HDL	1.94	1.30–2.88	3.26	0.0011	3.31	1.52–7.23	3.01	0.0026
TC/HDL	2.30	1.60–3.31	4.48	<0.0001	5.64	2.17–14.71	3.54	0.0004
LDL/HDL	2.20	1.49–3.26	3.94	0.0001	4.33	1.94–9.69	3.81	0.0004
TyG index	2.05	1.34–3.13	2.65	0.0079	4.99	1.59–15.63	2.76	0.0058

## Data Availability

Data are available from the corresponding author, Y.A., upon reasonable request and with permission of Prince Sultan Military Medical City (PSMMC).
